# Assessing gender differences in food preferences and physical activity: a population-based survey

**DOI:** 10.3389/fnut.2024.1348456

**Published:** 2024-02-20

**Authors:** Alessandra Feraco, Andrea Armani, Isaac Amoah, Elena Guseva, Elisabetta Camajani, Stefania Gorini, Rocky Strollo, Elvira Padua, Massimiliano Caprio, Mauro Lombardo

**Affiliations:** ^1^Department of Human Sciences and Promotion of the Quality of Life, San Raffaele Open University, Rome, Italy; ^2^Laboratory of Cardiovascular Endocrinology, San Raffaele Research Institute, IRCCS San Raffaele Roma, Rome, Italy; ^3^Department of Biochemistry and Biotechnology, Kwame Nkrumah University of Science and Technology, Kumasi, Ghana

**Keywords:** food preferences, dietary patterns, public health, training, eating habits, eating behaviors

## Abstract

**Introduction:**

Food preferences are influenced by various factors, such as culture, age, and gender. The relationship between food tastes, meal preferences, and eating habits has been studied extensively in recent years; however, research on gender differences in these fields still needs to be addressed. The aim of this study was to investigate gender differences in food preferences and eating habits through self-administered questionnaires in a large Italian population sample.

**Methods:**

The online survey included questions on food tastes, meal preferences, eating habits, and sport involvement.

**Results:**

The results of the study underline significant gender-specific dietary tendencies among the 2198 participants (1314 females and 884 males, average age 41.1 ± 12.7 yrs). The majority of subjects were in the annual income range between €20,000 and €40,000. Our analysis reveals significant gender differences in dietary preferences and eating habits. Men prefer red and processed meat, with significantly higher consumption rates than women. Women, on the other hand, show a greater inclination towards vegetables, whole grains, tofu, and high-cocoa-content dark chocolate, aligning with healthier food choices. The study also found differences in eating behaviors, including the frequency of meals, snacking habits, and hunger patterns: women tend to eat more frequently and report higher levels of hunger in the morning, while men tend to skip snacks. Furthermore, differences extend to eating contexts, such as the speed of eating, eating out, and eating alone, with men more likely to eat quickly and dine out. Episodes of uncontrolled eating without hunger also differ, with women reporting these behaviors more frequently than men. In addition, the analysis of sports preferences showed distinct patterns, with a lower percentage of women playing sports and those who do play sports preferring endurance and strength training, while men prefer strength training and endurance sports.

**Discussion:**

These findings elucidate the complex interplay of biological, cultural, and gender-based factors in shaping dietary preferences and eating behaviors. In particular, our study reveals that gender dynamics significantly influence food choice and eating habits: women tend to choose healthier foods and eat regular meals, while men show preferences for specific tastes and meal-related behaviors. This analysis underscores the nuanced differences between male and female dietary patterns, influenced not only by inherent biological factors such as genetics and hormonal responses but also by societal norms and cultural contexts. Taken together, our results highlight the importance of integrating different perspectives, thus providing valuable insights into the development of public health strategies and tailored nutrition interventions aimed at chronic disease prevention.

## 1 Introduction

In recent decades, extensive research in human physiology has revealed gender diversity in various hormonal pathways and medical parameters, as well as in dietary preferences (1). Throughout human history, women have been responsible for procreation in a nutrient-poor environment. For this reason, they have probably been subjected to greater evolutionary pressure than men (2). Gender differences in terms of food reward circuitry have been found with specific neural responses to sensory stimuli, as well as to hormonal signals and cognitive biases (3). Accordingly, in different functional neuroimaging studies, women exhibited increased activation in the frontal, limbic, and striatal areas of the brain, in response to various food stimuli. The existence of gender-specific neural correlates of food stimuli may contribute to women’s greater vulnerability to obesity and disordered eating (4).

Understanding the differences in taste and eating habits between men and women is essential in order to develop personalized nutritional strategies and improve the prevention of cardiometabolic diseases. Previous studies have shown that men and women tend to prefer divergent tastes and consume different types of food. Women usually show higher consumption of fruits and vegetables, increased dietary fiber intake, and lower fat intake, aligning with overall healthier food choices, thus demonstrating greater motivation for weight control (5, 6).

On the other hand, less healthy food choices among men may be linked to poorer nutritional knowledge. Moreover, men and women display different attitudes towards hedonic eating. Interestingly, a greater tendency to choose high-calorie food has been demonstrated in men upon neural activity stimulation, compared to women, suggesting a different and gender-related neuro-cognitive reward pattern (5, 7). Accordingly, clinical studies have consistently highlighted differences between men and women in various aspects of eating behaviors and nutritional choices. Women tend to show greater dietary restraint, hunger traits, disinhibition, eating disorder-related behaviors, depression, and stress compared to men. Additionally, women often express higher appeal and familiarity with low-calorie foods (8). This inclination towards healthier food choices is further evident in the dietary preferences of young adults, where women’s selections align more closely with principles of healthy eating, including increased consumption of fruits, vegetables, and products with lower energy values (6). Nevertheless, the mechanisms underlying these different behaviors are still unclear. We should not overlook the fact that the impact of food marketing also varies across genders, with boys’ preferences being more influenced by advertising (9).

Men show a preference for high-fat, strongly flavored meals, often driven by the pleasure of consumption. These preferences are reflected in men’s behaviors, such as eating sweets in front of the TV and frequenting fast food outlets (10, 11).

This study specifically aims to elucidate the differences underlying gender-related eating behaviors and preferences, providing insights essential for developing personalized nutritional strategies. Moreover, this study aims to explore the relationship between dietary choices and sports engagement, examining how these aspects interact differently across genders, thereby influencing overall health and wellness.

## 2 Materials and methods

### 2.1 Subjects

The study encompassed a demographically diverse group of participants recruited from an obesity center in Rome, Italy. The inclusion criteria were defined as follows: participants were required to be patients aged over 20 years, capable of completing an online survey in Italian before their initial visit and willing to provide written informed consent. The study deliberately included a wide age range to capture a comprehensive perspective on dietary patterns across different age groups. Participants were required to complete an online survey in Italian before their initial visit and provided written informed consent for participation. Additional data collected included information on smoking habits and yearly income. The study’s procedures, including the consent form, were approved by the IRCCS San Raffaele ethics committee, under the registration number RP 23/13, ensuring compliance with the ethical standards of the Declaration of Helsinki and its amendments. Patient enrollment for the study started in May 2023, and by October 2023, a total of 2,388 surveys were registered.

### 2.2 Survey

Participants were asked to complete a questionnaire prior to the first visit. Subjects then responded to a test via any electronic device with Internet access, which took approximately 30 min to complete. By accessing the link, participants were given the option to consent or not to be involved in the study. The online survey was structured and self-administered. We did not perform a formal validation test, but the survey was comparable to other validated food taste questionnaires (12). All responses were registered anonymously. The questionnaire was divided into four parts and has already been used in our previous work (13). The first part of the test assessed the number of daily meals (including main meals and snacks), when one was hungriest during the day, the presence of binge eating or cravings and the quality of sleep. Patients were asked if they skip meals, if they eat distractedly (e.g., not sitting at the table), and if they eat quickly. Sleep quality was also assessed and, in the case of sleep disturbances, whether there was a complaint of falling asleep, early awakening or sleep interruptions. In the subsequent section of the questionnaire, participants’ preferences towards specific foods were investigated through the question, “Do you like the following foods?” with response options of “I like it,” “I do not like it,” and “I do not know.” This inquiry aimed to discern participants’ taste preferences for a range of foods, including cow’s milk, vegetable-based alternatives such as soya milk, low-fat and low-sugar yoghurt, fresh cheese, various meat types, processed meats such as ham, fish, eggs, legumes, cooked and raw vegetables, fruits, different grains such as spelt and barley, foods containing wholemeal flour, dried fruits, tofu (soya), and dark chocolate with a cocoa content exceeding 75%. The subsequent section of the survey further explored participants’ favorite meals, their 24-h dietary recall and the frequency of consuming water, alcoholic beverages and sugary drinks. Lastly, the final segment of the questionnaire evaluated the presence or absence of physical activity and further assessed the number of weekly hours dedicated to sports activities (if applicable, categorized as <5 h, 5–10 h or > 10 h per week). Additionally, this section captured the timing of these activities during the day and the specific type of sport in which participants engaged.

### 2.3 Statistical analysis

Statistical analyses were conducted using SPSS v. 28 (IBM Corporation, Armonk, NY, USA). Descriptive statistics, including frequencies, were utilized to delineate the tastes and food consumption patterns of participants, analyzed collectively and stratified by gender. The chi-square test for independence was employed to ascertain differences in the consumption of various food items between genders, with the significance level set at *p* ≤ 0.05. For continuous variables, independent t-tests were applied to determine the statistical significance of differences between the male and female groups, while chi-square tests were used for categorical variables, including smoking status and income level. These tests provided *p*-values indicating the significance level for each compared variable.

## 3 Results

The study included a cohort of 2,198 participants, 1,314 women and 884 men (Table 1). The average age was 41.1 ± 12.7 years.

**Table 1:** Distribution of demographic variables by gender, smoking status, and income category in the study sample.

			Total	F	M	*p*
	Subjects’	*n* (%)	2,198	1,314 (59.8)	884 (40.2)	<0.001
Age	Age	yrs	41.1 ± 12.7	41.9 ± 12.9	40 ± 12.5	<0.001
	21–30	*n* (%)	451 (20.5)	247 (18.8)	204 (23.1)	0.006
	31–40	*n* (%)	648 (29.4)	369 (28)	279 (31.6)	
	41–50	*n* (%)	545 (24.8)	342 (26)	203 (23)	
	51–60	*n* (%)	343 (15.6)	214 (16.3)	129 (14.6)	
	61–70	*n* (%)	152 (6.9)	104 (7.9)	48 (5.4)	
	71–80	*n* (%)	54 (2.5)	33 (2.5)	21 (2.4)	
	>80	*n* (%)	5 (0.2)	5 (0.4)	0 (0)	<0.001
	Smokers	*n* (%)	539 (24.5)	329 (25.1)	210 (23.8)	0.45
Yearly income	<€20.000	*n* (%)	294 (13.4)	206 (15.7)	88 (10)	<0.001
	€20.000–€40.000	*n* (%)	1,530 (69.6)	891 (67.9)	639 (72.3)	
	€40.000- €60.000	*n* (%)	311 (14.2)	176 (13.4)	135 (15.3)	
	>€60.000	*n* (%)	63 (2.8)	41 (3.1)	22 (2.5)	

The table presents the distribution of various demographic data in the study sample, broken down by gender, smoking status, and income category, or continuous variables. The statistical significance of differences between the male and female groups was assessed using independent t-tests for continuous variables and chi-square tests for categorical variables such as smoking status and income category.

We observed that 24.5% of the participants were smokers, and there was no significant gender difference. Finally, the table categorizes the participants’ annual income, with a significant portion falling in the 20,000–40,000 €/yr. range.

**Figure 1:**
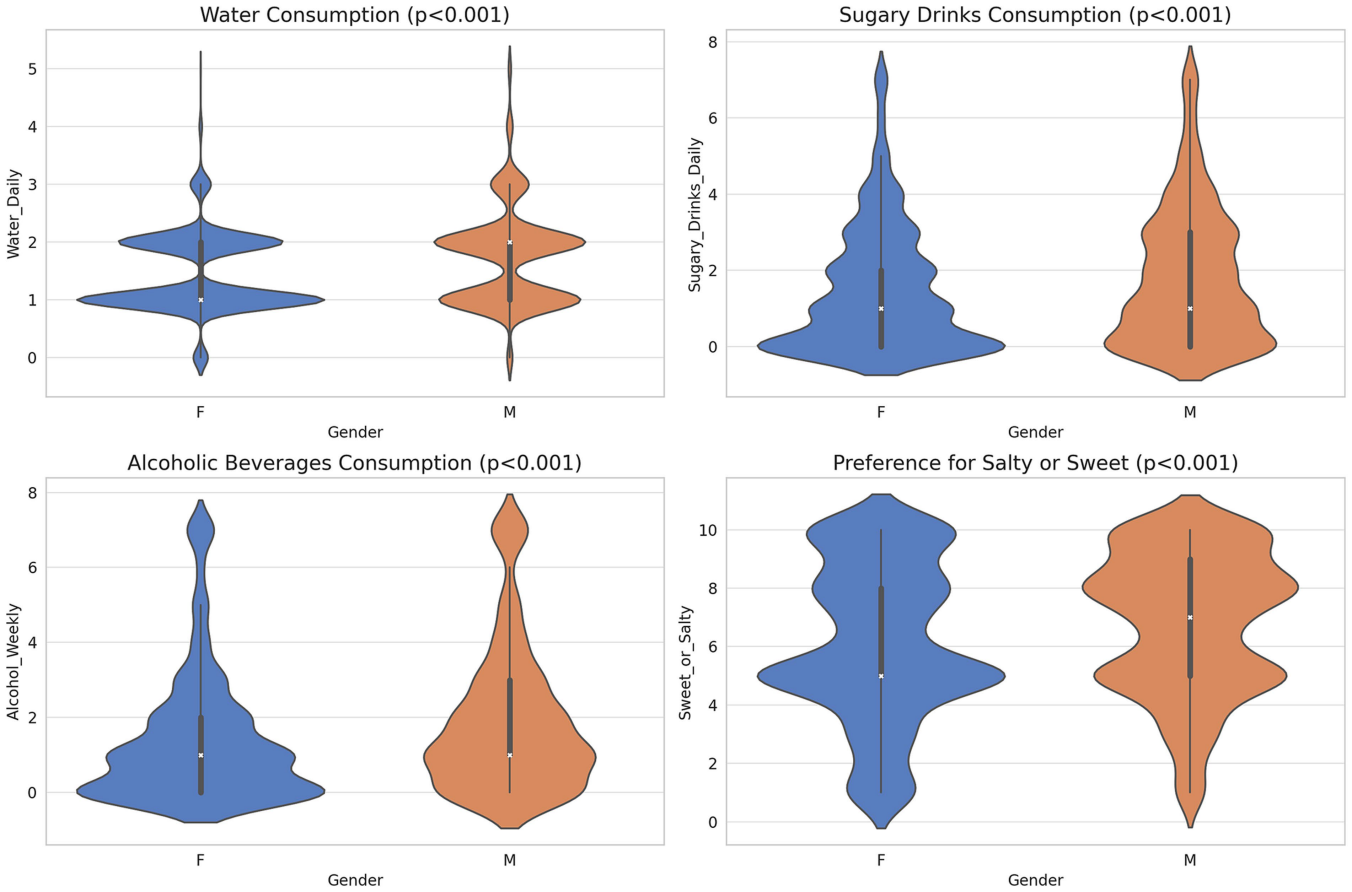
Gender differences in consumption habit preferences. The figure compares average daily water and sugary drink consumption, weekly alcoholic beverage consumption, and preference for sweet (1) or salty (10), between genders.

Figure 1 and Supplementary Table S1 present a comparison of consumption patterns by gender.

**Figure 2:**
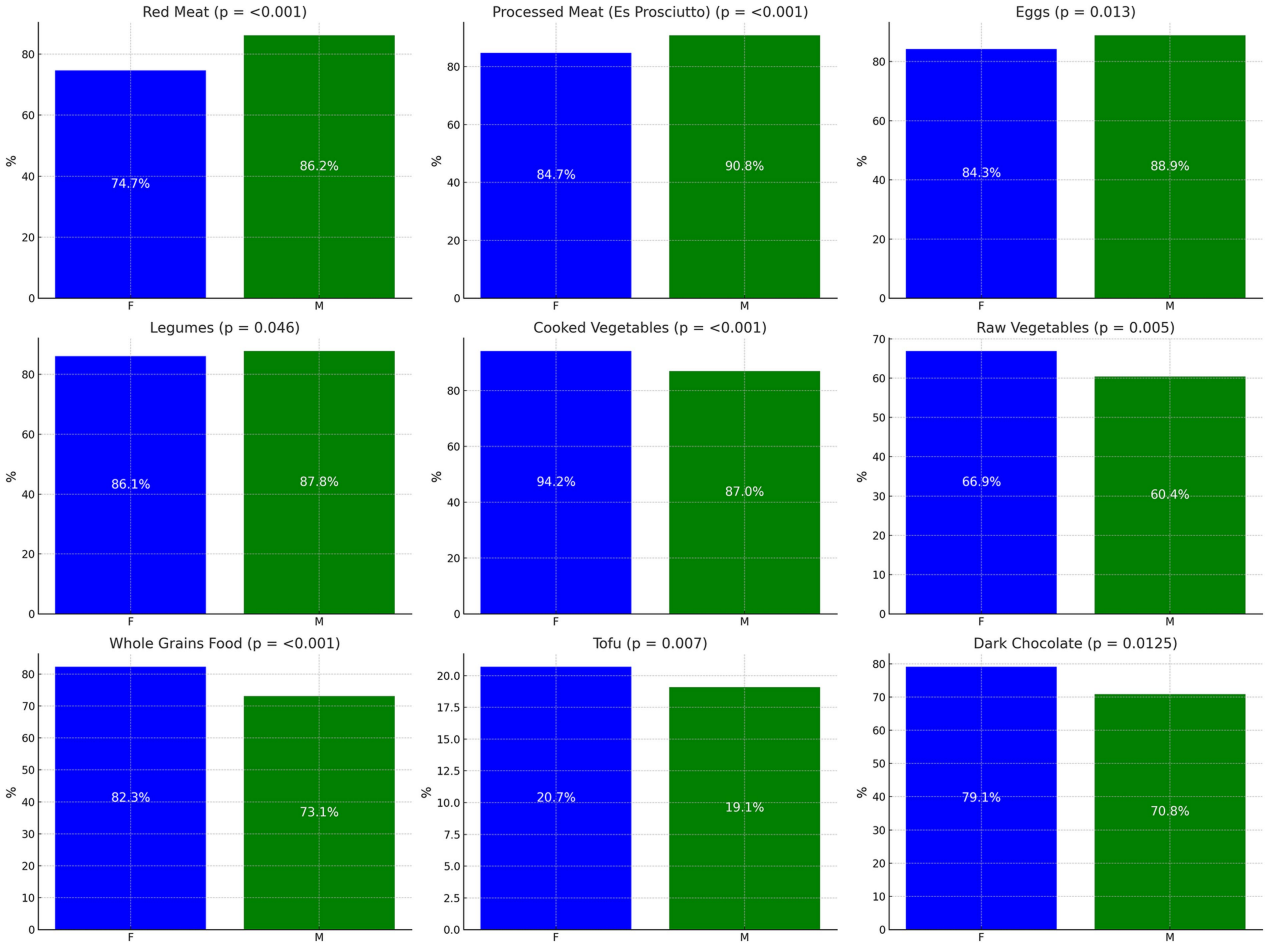
Comparative analysis of food preferences by gender. Comparative prevalence of ‘Yes’ responses indicating a preference for selected food items, distributed by gender. Percentages represent the proportion of respondents within each gender who favored the food item. *p*-values are indicated for each item. Statistical test: chi-square test.

Males and females reported an average daily water intake of 1.7 and 1.4 liters, respectively (*p* < 0.001). Males and females consumed sugary drinks or added sugar on average 1.8 and 1.4 times daily, respectively (*p* < 0.001). With regard to alcohol consumption, the average reported consumption was 2 times per week for men and 1.4 times for women (p < 0.001). The taste preference analysis revealed a mean score of 6.08 for females and a higher mean score of 6.9 for males, on a scale from sweet (1) to salty (10) (*t*-test; p < 0.001). Significant gender differences were noted in the preferences for nine food categories (Figure 2; Supplementary Table S2).

Our comprehensive analysis of dietary preferences by gender revealed distinct consumption patterns with statistical significance. Males exhibited a pronounced preference for red meat, with 86.2% reporting regular consumption compared to 74.7% of females, demonstrating a statistically significant difference (*p* < 0.001). This trend was similarly observed in the consumption of processed meats, such as prosciutto, where 90.8% of males vs. 84.7% of females reported consumption (p < 0.001).

By contrast, female participants showed a higher propensity to consume vegetables. Cooked vegetables were favored by 94.2% of females vs. 87.0% of males (*p* < 0.001), while raw vegetable consumption also proved to be more prevalent among females (66.9%) than males (60.4%, *p* = 0.005).

When it came to grain-based food choices, females were more inclined towards whole grains, with 82.3% consuming them regularly compared to 73.1% of males, indicating a significant difference (p < 0.001). Though not represented in the graphical analysis due to a borderline value of p, cereals such as spelt and barley showed a slight female preference (*p* = 0.0497).

The consumption of tofu and dark chocolate also highlighted gender-based preferences. Females were more likely to consume tofu (20.7% vs. 19.1%, *p* = 0.007) and dark chocolate with a higher cocoa content (79.1% vs. 70.8%, *p* = 0.0125). Notably, non-dairy alternatives such as soy milk were more popular among female participants (*p* = 0.025).

**Figure 3:**
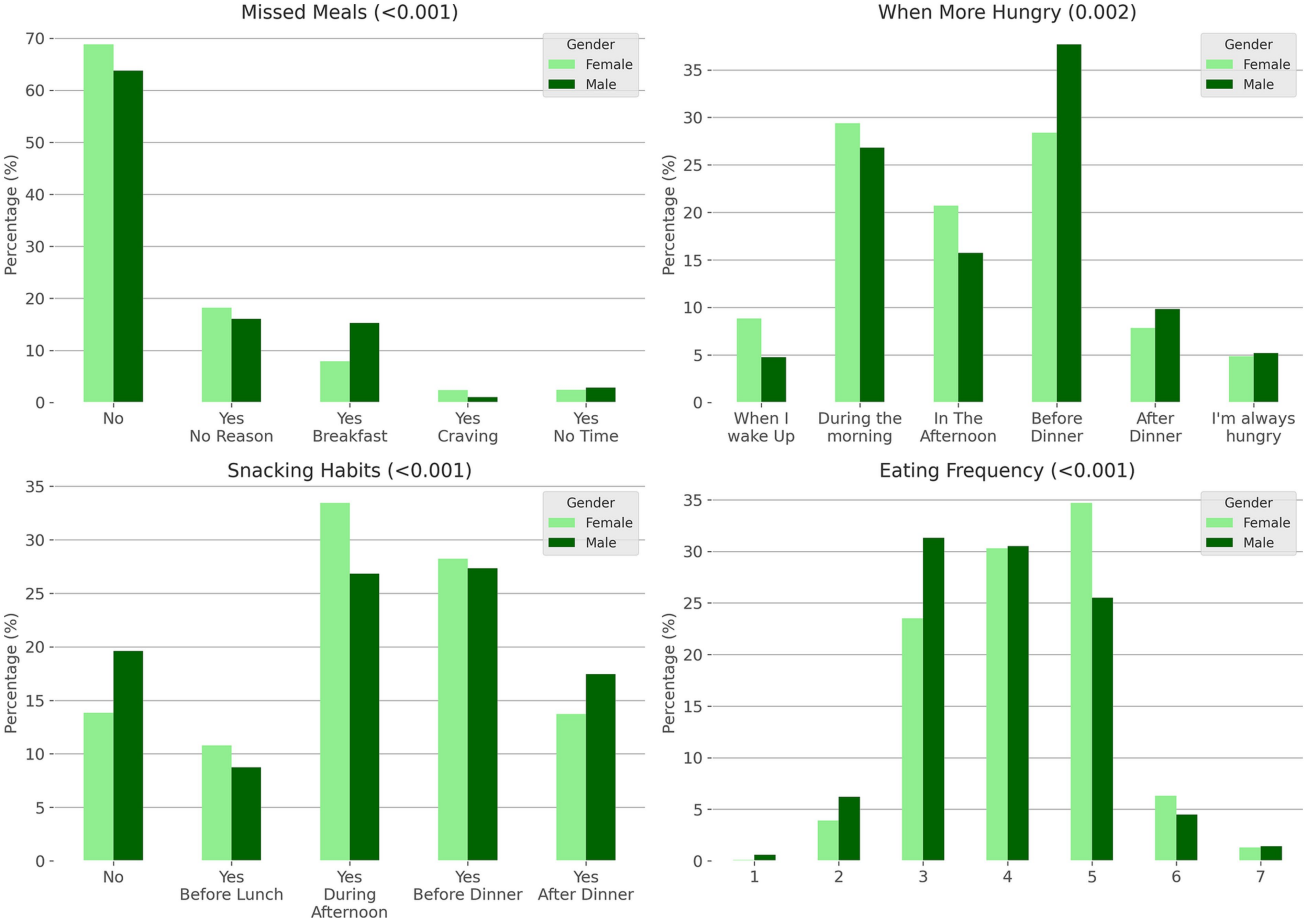
Differences in eating patterns and preferences by gender. The figure shows normalized histograms displaying the percentage of male and female participants’ responses to questions about missed meals, timing of hunger throughout the day, snacking habits, and number of daily meals (snacks included). Statistical significance was assessed using chi-square tests, with *p*-values indicating significant differences between genders for all categories.

The analysis of eating habits revealed significant differences between the genders in various aspects of eating behavior (Figure 3; Supplementary Table S3).

The survey of hunger patterns throughout the day showed that males felt hungrier before dinner than females (37.7% vs. 28.4%). By contrast, females reported feeling hungrier in the morning than males (29.4% vs. 26.8%). Snacking habits also varied significantly between genders. Females were more inclined to snack during the afternoon (33.5% vs. 26.9% of males). By contrast, the practice of not snacking between meals was more common among males (19.6% vs. 13.8% of females). Regarding the frequency of daily meals, the data showed that females tended to eat more frequently, with a notable percentage eating five times a day (34.7% for females vs. 25.5% for males).

**Figure 4:**
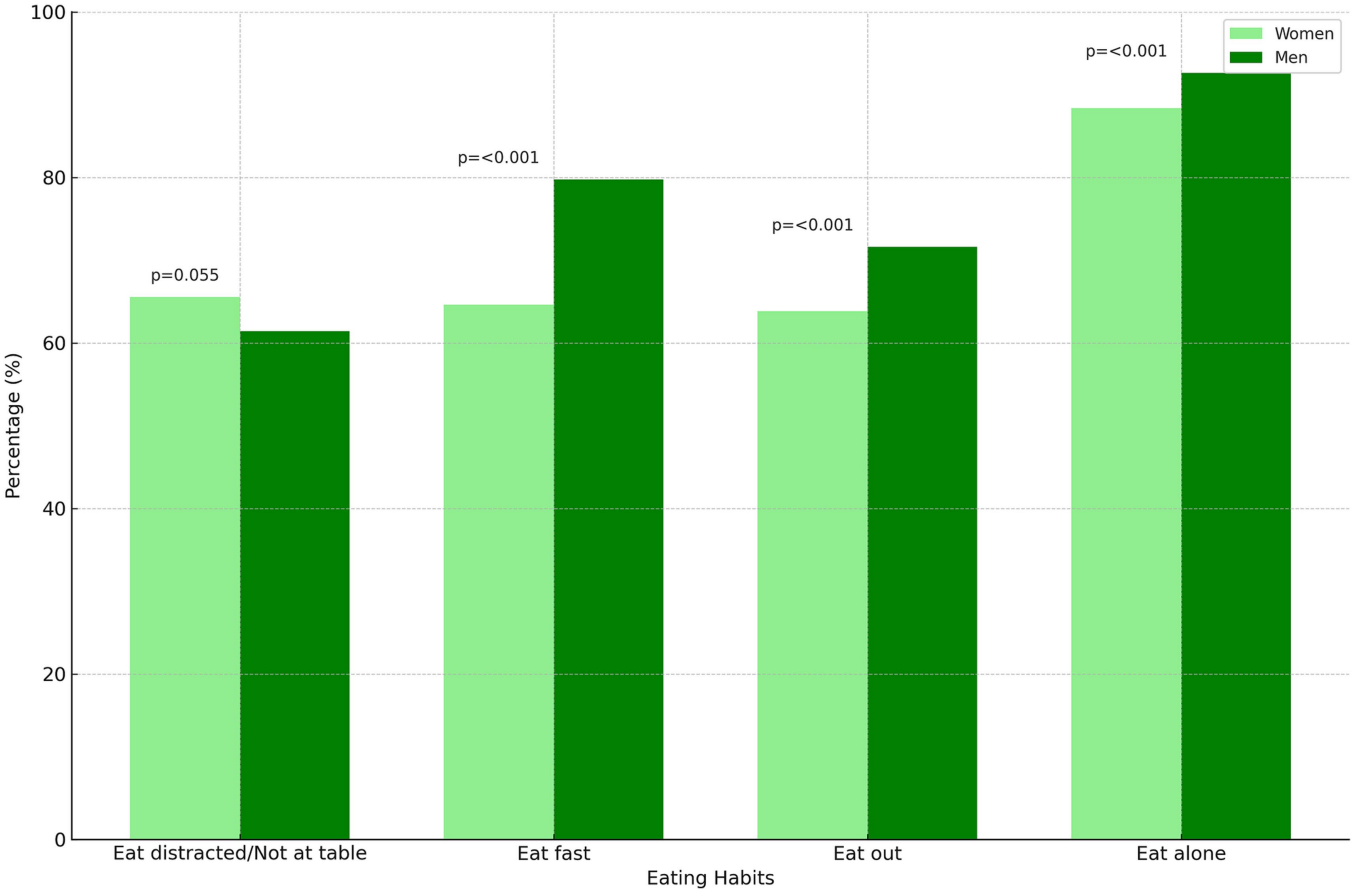
Gender differences in eating habits. Comparison of eating habits by gender, showing the percentage of women (in light green) and men (in dark green) who reported specific behaviors. The p-values indicate the statistical significance of the observed differences, suggesting distinct eating behaviors between genders.

In our results, we examined eating habits based on gender, focusing on four key behaviors: eating distracted or not at the table, eating fast, eating out, and eating alone (Figure 4). We used a chi-square test to assess the differences between the responses of men and those of women. For the question on eating distracted or not at the table, we found a slightly higher percentage of women (65.53%) than men (61.43%), with a value of p of 0.055. For the other questions, we observed statistically significant differences: eating fast (64.61% of women vs. 79.75% of men, *p* < 0.001), eating out (63.84% of women vs. 71.61% of men, p < 0.001), and eating alone (88.36% of women vs. 92.60% of men, p < 0.001).

**Figure 5:**
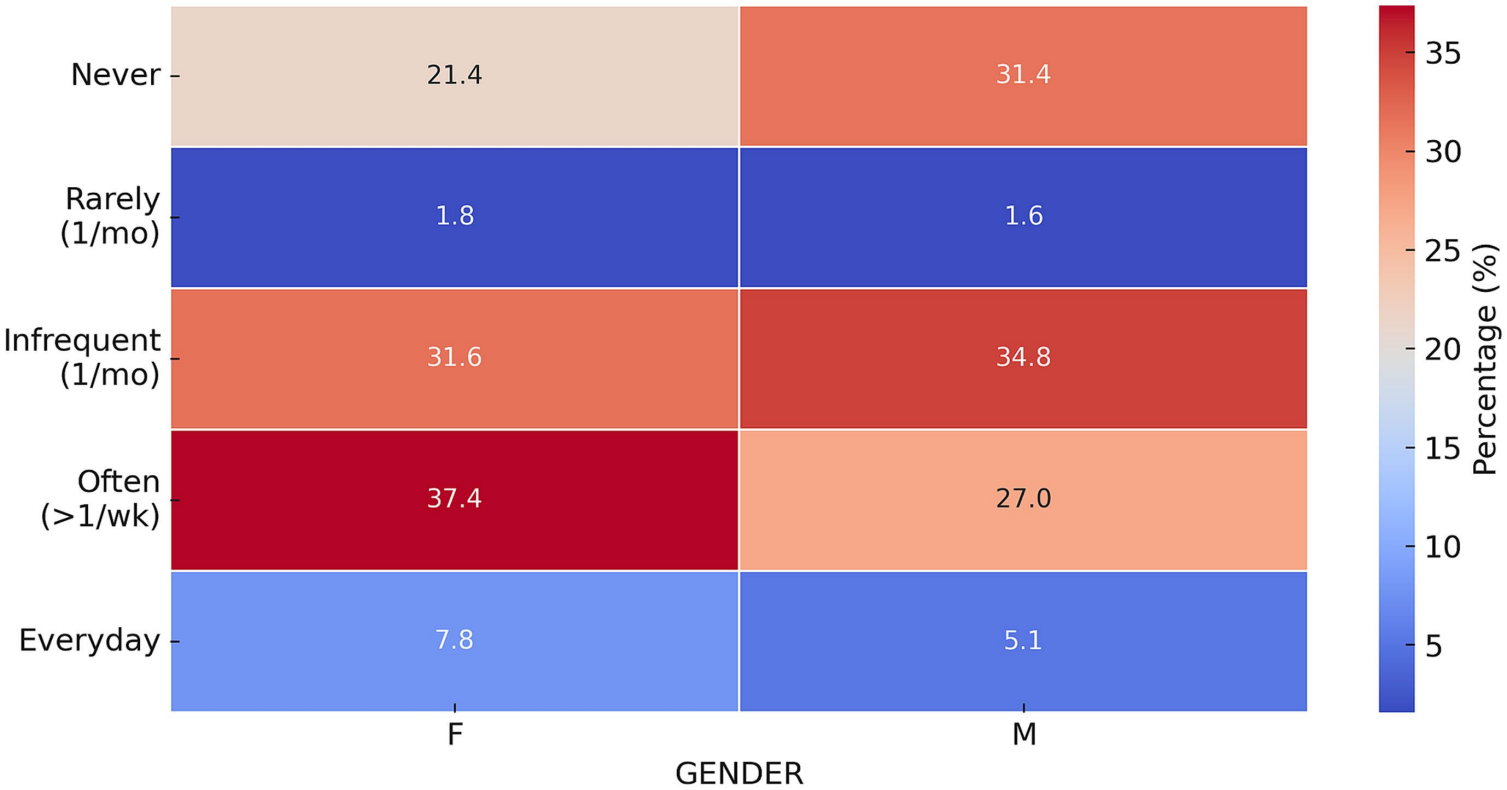
Normalized distribution of responses to the question “Do you happen to eat uncontrollably even if you’re not hungry?” by gender. Heatmap illustrating the normalized distribution of responses to the question “Do you happen to eat uncontrollably even if you are not hungry?” The responses are categorized by gender. The color gradient represents the percentage of respondents in each category, with annotations indicating the exact percentage. Statistical test: chi-square test, *p* < 0.001.

In our analysis, we examined the frequency of uncontrolled eating episodes despite the absence of hunger, stratified by gender (Figure 5).

The analysis of uncontrolled eating habits between genders revealed significant differences (p < 0.001) in the answers given. Women reported eating uncontrollably less frequently every day (7.8%) than men (5.1%), while a higher percentage of women (37.4%) than men (27.0%) indicated that they eat uncontrollably more than once a week. By contrast, a higher percentage of men (31.4%) than women (21.4%) responded that they never eat uncontrollably.

In our study, we examined sports preferences divided by gender, including four main categories (Supplementary Table S4): Endurance Sports, Skill Sports, Strength Training, and Team Sports, as well as those who do not participate in sports. The data collected showed that a significant proportion of participants, particularly females (52.4%), do not participate in sports. Among males, this percentage stands at 41.9%. Amongst females practicing sports, Endurance Sports were the most popular (26.9%), followed by Strength Training (24.6%). Skill Sports and Team Sports attracted less interest. As for males, Strength Training emerged as the most popular category (32.2%), with Endurance Sports following closely behind. It is important to note that the percentages exceed 100% because individuals may participate in more than one sport, thus being counted in more than one category.

## 4 Discussion

Several preclinical and clinical studies have investigated the genetic basis of taste perception and metabolism, demonstrating that these factors contribute to the diversity of food choices among individuals (14–17). Of note, the COVID-19 pandemic has significantly influenced dietary choices and physical activity, altering food habits and preferences, as well as the frequency and types of exercise (18–20). Determinants of dietary habits extend to encompass the significant influence of gender on dietary choices. In fact, gender dynamics influence not only food preference but also individuals’ overall dietary nutritional behaviors. In the last 10 years, several clinical studies have evaluated gender-specific patterns in food selection, revealing the existence of differences in nutrient intake and food choices between males and females (21–24).

In the present study, we demonstrated significant gender differences in eating patterns, taste preferences, and meal-related behaviors. These differences are not only biologically intrinsic but may also be influenced by the cultural context in which individuals are raised and live (25). The greater preference for sweet and sour flavors among females, and for bitter and salty flavors among males, might be linked to evolutionary dietary roles, as well as current societal norms. Females’ propensity for breakfast regularity and higher fruit and vegetable intake can be tied to greater health consciousness, often socially encouraged among women (26, 27).

Cultural norms and gender roles influence dietary choices, with men consuming more red and processed meat while women prefer healthier foods (28). Understanding these gender-specific preferences may be critical for targeted public health interventions. For example, encouraging a plant-based balanced diet among males could potentially mitigate the health risks associated with high red meat consumption (29–31). Similarly, the promotion of alternative protein sources, such as foods derived from soy, as a substitute for meat and dairy products, could promote the improvement of the diets of both sexes, males in particular (32). The higher intake of red meat among males vs. females aligns with a study by Ritzel and Mann, which identified a positive correlation between red meat consumption and perceived masculinity, evolving from infancy to late adulthood (33). Kemper et al. (34) suggested that social stigmas and personal preferences may make it difficult for some men to reduce meat intake. The motivations to avoid red and processed meat, as highlighted by Clonan et al., often are related to human health and animal welfare (35). However, being informed about the health and environmental benefits of reducing red meat consumption could motivate males’ dietary adjustments (36). Our study indicates that women generally choose more plant-based foods, particularly favoring cooked and raw vegetables, legumes, whole grains, tofu, and dark chocolate with a high cocoa content. This trend is in line with previous studies showing gender differences in food preferences, with women often opting for foods that are considered healthier (37, 38).

A gender-based comparison of eating behaviors revealed several key differences that warrant discussion. Firstly, the tendency of men to skip meals, particularly breakfast, more frequently than women could indicate different lifestyle patterns or stressors between genders, suggesting gender differences in emotional or psychological responses to food. The distribution of hunger throughout the day also differs significantly between genders. Men reported increased hunger before dinner, whereas women experienced a more even distribution of hunger. In a study by Bédard et al. (39), gender differences were also evident in appetite sensation responses to Mediterranean diet (MD) meals. While both genders experienced increased hunger and appetite over time on the MD, only men reported an increase in prospective food consumption before meals. Leone et al. provide further evidence supporting the hypothesis that appetite regulation differs between men and women, particularly in relation to postprandial ghrelin regulation (40). Their study found that after consuming a balanced mixed meal, women experience greater satiety immediately, while men show a delayed suppression of hunger. This difference is attributed to a smaller decrease in postprandial ghrelin in men.

Our findings on snacking habits reveal that men are hungrier in the second part of the day than in the morning but tend to snack less than women. Men generally report the highest levels of hunger before dinner, while women experience peak hunger in the early afternoon, often accompanied by cravings. This observation is in accordance with research by Hartmann et al., who found that a high snack frequency can be associated with both healthy and unhealthy dietary behaviors (41). Women tend to make healthier dietary choices, often opting for fruits as snacks, whereas men are more inclined towards unhealthy options such as sweets and savories. Further, Hunter and Mattes note that the prevalence of snacking has increased in Western nations over the past 35 years, with a positive association between eating frequency and energy intake (42). However, the impact of this increase on obesity is complex. Snacking can contribute to higher energy intake, especially when snacks are unplanned and evoke weak compensatory responses in terms of reduced energy intake at subsequent meals (43). Factors contributing to this include weak satiation/satiety effects, distracted eating, a low thermogenic response, and disrupted biological cycles. Notably, the issue with snacks is not necessarily their energy density, as even healthier options such a fruits and vegetables can increase total energy intake. The key is how snacks are incorporated into the diet (44). When balanced within an energy-controlled diet, snacking can aid in managing eating schedules, improving nutrient quality, and potentially moderating blood sugar and metabolic disease risk factors (45). These insights suggest that the differences in snacking habits between men and women, as observed in the study, could reflect broader dietary patterns and lifestyle choices, with implications for overall energy intake and, potentially, body weight management.

Our study offers an interesting perspective on the differences in eating habits between the sexes. The data collected indicate that men tend to eat more often alone, away from home, and quickly. This behavior could be related to a greater work commitment among men, who show the greatest hunger peak upon returning home, in the pre-dinner period. In a recent scoping review conducted to characterize snacks and snacking habits (46), afternoon snacking emerged as a compromise between taste, need, hunger, health, convenience, and weight control and was further characterized by distracted eating. This type of afternoon snacking is associated with individuals with a higher body mass index, obesogenic dietary patterns, and those who are less health-conscious and less concerned about weight management. The distinction between male and female eating styles highlighted by our study underlines the importance of considering gender dynamics when investigating eating habits and their implications for health. Understanding these differences can be crucial for the development of more effective and personalized nutrition intervention and health promotion strategies.

**Figure 6:**
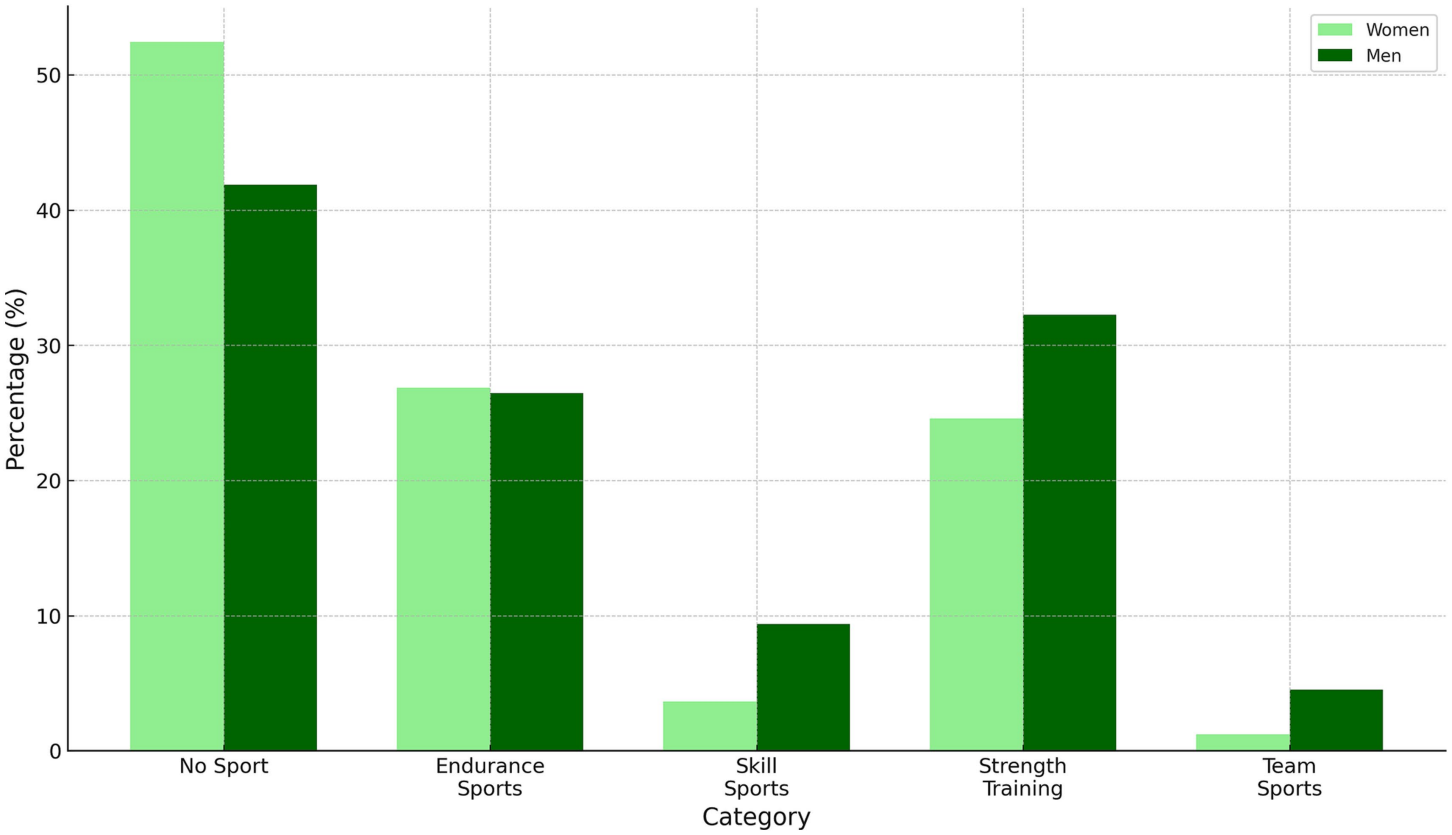
Comparative analysis of sports preferences by gender in different categories. The graph represents the distribution of sports preferences among male and female participants, categorized into Endurance Sports, Skill Sports, Strength Training, and Team Sports, based on a revised classification (see Supplementary Table S1 for detailed categorization of sports activities). The percentages exceed 100% because some individuals practice more than one sport and were thus counted in more than one category.

In this study, a marked gender disparity in sports participation was evident: men reported much higher levels of participation in sport than women. Further insight is provided by Figure 6, which categorizes sports activities into Endurance Sports, Skill Sports, Strength Training, and Team Sports. The data indicate a preference for Strength Training and Team Sports among men. The observed trends in sports preferences not only reflect gender-based differences in physical activity choices but also suggest underlying physiological, psychological, and sociocultural factors at play. Men’s predilection for strength training and team sports might be attributed to societal norms valorizing physical strength and competitive team dynamics in male socialization (47–49); on the other hand, women’s propensity for endurance and skill sports may stem from biological aspects such as lean body mass distribution and societal influences that favor activities associated with precision and endurance (50, 51). These nuances in sports participation patterns necessitate a multifaceted approach in public health policies (52), where interventions are designed with an acute awareness of these gender-specific predilections and the complex of factors influencing them, thereby ensuring more effective and inclusive promotion of physical activity across genders (53–55).

The results of our study offer significant implications for clinical practice, especially in the field of personalized nutrition and chronic disease prevention. The clear evidence of gender-based differences in dietary behavior and preferences suggests the need for targeted nutrition strategies that take these variations into account. For example, interventions could include the development of separate nutrition education programs and guidelines specifically targeting men and women, reflecting their specific dietary needs and habits. For men, who eat more often alone, outside the home, and in a hurry, strategies could focus on promoting the choice of healthier fast food options, the importance of not skipping meals, especially breakfast, and ways to incorporate quick and nutritious meals into a busy lifestyle. For women, who may have different nutritional needs and show a preference for afternoon snacks, interventions could emphasize the importance of balanced, nutrient-rich snacks that satisfy hunger without contributing to obesogenic eating patterns. Furthermore, understanding these gender differences allows health professionals to better tailor advice for weight management and chronic disease prevention. For example, men could benefit from targeted advice on reducing portion sizes and choosing nutrient-rich foods to mitigate the effects of frequent eating out, while women could receive guidance on how to manage cravings and make healthier snack choices. Incorporating gender-specific considerations into dietary recommendations could improve the effectiveness of public health initiatives aimed at reducing the prevalence of chronic diseases such as obesity, diabetes, and cardiovascular disease. Tailored interventions could also address the psychological and social factors that influence dietary behavior, further improving the success of nutrition-related health promotion initiatives.

This study has limitations. Firstly, the use of self-reported data in the survey may introduce a bias, as participants may not accurately recall or may choose to selectively report their eating habits and physical activities. This bias could potentially skew the results, especially in areas involving sensitive topics such as eating disorders or the consumption of high-calorie foods. It is important to emphasize that although the test was conducted online, it was always discussed with a nutrition professional. Another limitation concerns the sample population, which was taken from an obesity center in Rome. This population may not be representative of the general population, especially in terms of eating habits and health awareness. The results, therefore, may have limited applicability to larger populations with different cultural, socioeconomic, or health backgrounds. We acknowledge that recruiting participants from a specialized obesity center may introduce a bias that limits the generalizability of our findings to the general population. Research indicates substantial differences in taste function and ingestive behaviors between obese and non-obese individuals, which could influence dietary choices and perceptions uniquely within our study cohort (56–58). These variations highlight the intricate relationship between obesity and taste perception, emphasizing the need for caution when extending our results beyond the specific context of this study. The limitation related to the lack of formal validation of the survey instrument, although noteworthy, may be somewhat mitigated by several factors. Firstly, the structure and content of the survey were modelled on other validated food taste questionnaires, providing a basis for its reliability. This approach ensures that the survey questions are based on established research and methodologies, which is likely to increase the accuracy and relevance of the data collected. Furthermore, although no specific validation was conducted for this particular survey, the use of established question types and formats drawn from similar validated instruments adds a degree of credibility to the results. However, it is important to recognize that the absence of a specific validation process could introduce some uncertainty regarding the accuracy and consistency of the responses. Future studies could benefit from formal validation of the survey instrument, which would further strengthen the results and contribute to the robustness of research in this field.

## 5 Conclusion

The study highlights important gender differences in eating and sports behavior, underlining the critical role of gender in the development of nutritional strategies and the prevention of chronic diseases. The research highlights the need for gender differentiated approaches to promote healthy eating habits. The research opens new avenues for personalized nutritional interventions, encouraging health professionals and policy makers to develop targeted strategies that take into account the specific needs of different genders. This could include educational programs and marketing campaigns tailored to the dietary trends and health concerns of men and women, promoting a more balanced and conscious approach to nutrition. This direction could lead to better health outcomes and a higher quality of life across genders. Further research is needed to explore the factors influencing gender differences in eating habits and sport, emphasizing the importance of considering cultural, social, and biological aspects.

## Data availability statement

The original contributions presented in the study are included in the article/Supplementary material, further inquiries can be directed to the corresponding author.

## Ethics statement

The studies involving humans were approved by Comitato Etico IRCCS San Raffaele Roma. The studies were conducted in accordance with the local legislation and institutional requirements. Written informed consent for participation in this study was provided by the participants' legal guardians/next of kin.

## Author contributions

AF: Conceptualization, Investigation, Writing – original draft. AA: Writing – review & editing, Funding acquisition, Supervision, Validation. IA: Formal analysis, Investigation, Software, Writing – original draft. EG: Investigation, Software, Data curation, Writing – original draft. EC: Data curation, Investigation, Writing – review & editing. SG: Resources, Writing – review & editing. RS: Resources, Writing – review & editing, Supervision, Visualization. EP: Project administration, Writing – original draft. MC: Project administration, Funding acquisition, Methodology, Writing – review & editing. ML: Writing – review & editing, Conceptualization, Data curation, Writing – original draft.
