# Supplementary material for: Assessing gender differences in food preferences and physical activity: a population-based survey
Source: Front Nutr. 2024 Feb 20;11:1348456. doi: 10.3389/fnut.2024.1348456 (PMC10912473; doi:10.3389/fnut.2024.1348456)
Supplement: Supplementary file 1 [file Data_Sheet_1.docx]

**Table 1s** - Differences in drinking habits

|  |  | F | | M | | p |
| --- | --- | --- | --- | --- | --- | --- |
|  |  | n. | % | n. | % |  |
| How Many Litres Of Water Do You Drink Per Day on average? | 0.0 | 39 | 3 | 14 | 1,6 | <0.001 |
|  | 1.0 | 738 | 56,2 | 366 | 41,4 |  |
|  | 2.0 | 476 | 36,2 | 390 | 44,1 |  |
|  | 3.0 | 55 | 4,2 | 94 | 10,6 |  |
|  | 4.0 | 5 | 0,4 | 15 | 1,7 |  |
|  | 5.0 | 1 | 0,1 | 5 | 0,6 |  |
| How Many Sugary Drinks or added sugar do You Consume Per Day on average? | 0.0 | 498 | 37,9 | 274 | 31 | <0.001 |
|  | 1.0 | 277 | 21,1 | 187 | 21,2 |  |
|  | 2.0 | 225 | 17,1 | 130 | 14,7 |  |
|  | 3.0 | 157 | 11,9 | 141 | 16 |  |
|  | 4.0 | 93 | 7,1 | 78 | 8,8 |  |
|  | 5.0 | 30 | 2,3 | 33 | 3,7 |  |
|  | 6.0 | 12 | 0,9 | 18 | 2 |  |
|  | 7.0 | 22 | 1,7 | 23 | 2,6 |  |
| How Many Times Do You Consume Alcoholic Beverages In A Week? | 0.0 | 496 | 37,7 | 214 | 24,2 | <0.001 |
|  | 1.0 | 376 | 28,6 | 236 | 26,7 |  |
|  | 2.0 | 196 | 14,9 | 178 | 20,1 |  |
|  | 3.0 | 105 | 8 | 101 | 11,4 |  |
|  | 4.0 | 36 | 2,7 | 55 | 6,2 |  |
|  | 5.0 | 29 | 2,2 | 29 | 3,3 |  |
|  | 6.0 | 15 | 1,1 | 4 | 0,5 |  |
|  | 7.0 | 61 | 4,6 | 67 | 7,6 |  |
| Do you Like Sweet (1) Or Salty (10)? | 1 | 102 | 7,8 | 23 | 2,6 | < 0.001 |
|  | 2 | 44 | 3,3 | 14 | 1,6 |  |
|  | 3 | 89 | 6,8 | 42 | 4,8 |  |
|  | 4 | 74 | 5,6 | 47 | 5,3 |  |
|  | 5 | 353 | 26,9 | 181 | 20,5 |  |
|  | 6 | 77 | 5,9 | 52 | 5,9 |  |
|  | 7 | 96 | 7,3 | 81 | 9,2 |  |
|  | 8 | 180 | 13,7 | 188 | 21,3 |  |
|  | 9 | 90 | 6,8 | 111 | 12,6 |  |
|  | 10 | 209 | 15,9 | 145 | 16,4 |  |

n: number of subjects

**Table 2s** - Differences in food preferences

|  |  | F | | M | | p |
| --- | --- | --- | --- | --- | --- | --- |
|  |  | n. | % | n. | % |  |
| Vegetable Drinks  (Eg  Soy  Milk) | Don't know | 67 | 5,1 | 63 | 7.1 | 0.061 |
|  | No | 519 | 39,5 | 358 | 40.5 |  |
|  | Sometimes | 252 | 19,2 | 182 | 20.6 |  |
|  | Yes | 476 | 36,2 | 281 | 31.8 |  |
| Red Meat | Don't know | 7 | 1.1 | 3 | 0.7 | <0.001 |
|  | No | 66 | 10 | 28 | 6 |  |
|  | Sometimes | 94 | 14.2 | 33 | 7.1 |  |
|  | Yes | 494 | 74.7 | 401 | 86.2 |  |
| Processed  Meat  (Es  Prosciutto) | Don't know | 4 | 0,3 | 5 | 0,6 | <0.001 |
|  | No | 103 | 7,8 | 43 | 4,9 |  |
|  | Sometimes | 94 | 7,2 | 33 | 3,7 |  |
|  | Yes | 1113 | 84,7 | 803 | 90,8 |  |
| Eggs | Don't know | 2 | 0,2 | 1 | 0,1 | 0.013 |
|  | No | 65 | 4,9 | 24 | 2,7 |  |
|  | Sometimes | 139 | 10,6 | 73 | 8,3 |  |
|  | Yes | 1108 | 84,3 | 786 | 88,9 |  |
| Legumes | Don't know | 1 | 0,1 | 2 | 0,2 | 0.046 |
|  | No | 60 | 4,6 | 21 | 2,4 |  |
|  | Sometimes | 122 | 9,3 | 85 | 9,6 |  |
|  | Yes | 1131 | 86,1 | 776 | 87,8 |  |
| Cooked  Vegetables | Don't know | 1 | 0,1 | 2 | 0,2 | <0.001 |
|  | No | 18 | 1,4 | 20 | 2,3 |  |
|  | Sometimes | 57 | 4,3 | 93 | 10,5 |  |
|  | Yes | 1238 | 94,2 | 769 | 87 |  |
| Raw  Vegetables | Don't know | 123 | 9,4 | 88 | 10 | 0.005 |
|  | No | 168 | 12,8 | 125 | 14,1 |  |
|  | Sometimes | 144 | 11 | 137 | 15,5 |  |
|  | Yes | 879 | 66,9 | 534 | 60,4 |  |
| Cereals  (Eg  Spelled,  Barley) | Don't know | 4 | 0,3 | 6 | 0,7 | 0.133 |
|  | No | 72 | 5,5 | 55 | 6,2 |  |
|  | Sometimes | 111 | 8,4 | 96 | 10,9 |  |
|  | Yes | 695 | 52,9 | 450 | 50,9 |  |
| Whole  Grains  Food | Don't know | 3 | 0,2 | 11 | 1,2 | < 0.001 |
|  | No | 73 | 5,6 | 56 | 6,3 |  |
|  | Sometimes | 157 | 11,9 | 171 | 19,3 |  |
|  | Yes | 1081 | 82,3 | 646 | 73,1 |  |
| Tofu | Don't know | 114 | 8,7 | 112 | 12,7 | 0.007 |
|  | No | 620 | 47,2 | 378 | 42,8 |  |
|  | Sometimes | 308 | 23,4 | 225 | 25,5 |  |
|  | Yes | 272 | 20,7 | 169 | 19,1 |  |
| Cow’s  Milk | Don’t know | 14 | 1,1 | 5 | 0,6 | 0.63 |
|  | No | 325 | 24,7 | 227 | 25,7 |  |
|  | Sometimes | 165 | 12,6 | 110 | 12,4 |  |
|  | Yes | 810 | 61,6 | 542 | 61,3 |  |
| Low-Fat  Low-Sugar  Yogurt | Don't know | 7 | 0,5 | 5 | 0,6 | 0.116 |
|  | No | 273 | 20,8 | 151 | 17,1 |  |
|  | Sometimes | 287 | 21,8 | 220 | 24,9 |  |
|  | Yes | 747 | 56,8 | 508 | 57,5 |  |
| Fruits | Don't know | 1 | 0,1 | 1 | 0,1 | 0.744 |
|  | No | 31 | 2,4 | 15 | 1,7 |  |
|  | Sometimes | 111 | 8,4 | 77 | 8,7 |  |
|  | Yes | 1171 | 89,1 | 791 | 89,5 |  |
| Fish | Don't know | 6 | 0,5 | 4 | 0,5 | 0.559 |
|  | No | 55 | 4,2 | 38 | 4,3 |  |
|  | Sometimes | 155 | 11,8 | 87 | 9,8 |  |
|  | Yes | 1098 | 83,6 | 755 | 85,4 |  |
| Fresh  Cheeses | Don't know | 3 | 0,2 | 2 | 0,2 | 0.966 |
|  | No | 115 | 8,8 | 83 | 9,4 |  |
|  | Sometimes | 86 | 6,5 | 58 | 6,6 |  |
|  | Yes | 1110 | 84,5 | 741 | 83,8 |  |
| Nuts | Don't know | 2 | 0,2 | 5 | 0,6 | 0.287 |
|  | No | 76 | 5,8 | 52 | 5,9 |  |
|  | Sometimes | 89 | 6,8 | 69 | 7,8 |  |
|  | Yes | 1147 | 87,3 | 758 | 85,7 |  |
| Dark Chocolate | Don't know | 5 | 7.3 | 6 | 1.2 | 0.013 |
|  | No | 78 | 11.4 | 72 | 15.3 |  |
|  | Sometimes | 59 | 8.6 | 60 | 12.7 |  |
|  | Yes | 538 | 79.1 | 334 | 70.8 |  |

n: number of subjects

Table  3s - Relationship with meals, sleep habits and sports frequency differences

|  |  |  | F | | M | | p |
| --- | --- | --- | --- | --- | --- | --- | --- |
|  |  |  | n. | % | n. | % |  |
| Do You Ever Miss Meals? | NO |  | 904 | 68.8 | 564 | 63.8 | <0.001 |
|  | YES |  | 234 | 17.8 | 137 | 15.5 |  |
|  |  | I often skip meals because I don't have time | 4 | 0.3 | 5 | 0.6 |  |
|  |  | I often skip meals because I snack between meals | 1 | 0.1 | 0 | 0 |  |
|  |  | Yes, but only breakfast | 104 | 7.9 | 135 | 15.3 |  |
|  |  | Yes for craving | 31 | 2.4 | 9 | 1 |  |
|  |  | I have no time | 32 | 2.4 | 25 | 2.8 |  |
| When Are You More Hungry During The Day? |  | When I wake Up | 116 | 8.8 | 42 | 4.8 | 0.002 |
|  |  | During the morning | 386 | 29.4 | 237 | 26.8 |  |
|  |  | In The Afternoon | 272 | 20.7 | 139 | 15.7 |  |
|  |  | Before Dinner | 373 | 28.4 | 333 | 37.7 |  |
|  |  | After Dinner | 103 | 7.8 | 87 | 9.8 |  |
|  |  | I'm always hungry | 64 | 4.9 | 46 | 5.2 |  |
| Do you snack between meals? |  | No | 122 | 13.8 | 119 | 19.6 | <0.001 |
|  |  | Yes, before lunch | 95 | 10.8 | 53 | 8.7 |  |
|  |  | Yes, during the afternoon | 295 | 33.5 | 163 | 26.9 |  |
|  |  | Yes, before dinner | 249 | 28.2 | 166 | 27.4 |  |
|  |  | Yes, after dinner | 121 | 13.7 | 106 | 17.5 |  |
| How Many Times Do You Eat A Day? |  | 1 | 1 | 0.1 | 5 | 0.6 | <0.001 |
|  |  | 2 | 51 | 3.9 | 55 | 6.2 |  |
|  |  | 3 | 308 | 23.5 | 277 | 31.3 |  |
|  |  | 4 | 398 | 30.3 | 270 | 30.5 |  |
|  |  | 5 | 456 | 34.7 | 225 | 25.5 |  |
|  |  | 6 | 82 | 6.3 | 40 | 4.5 |  |
|  |  | 7 | 17 | 1.3 | 12 | 1.4 |  |

**Table 4s - Categorization of Sports into Endurance, Skill, Strength, and Team-Based Activities**

| **Endurance Sports** | **Skill Sports** | **Strength Training** | **Team Sports** |
| --- | --- | --- | --- |
| Cardio | Boxing | Bodybuilding | Basketball |
| Fitness | Classical Dance | Callisthenics | Football |
| Running | Climbing | Canoeing | Handball |
| Spinning | Dance | Crossfit | Hockey |
| SUP | Fighting | Functional Training | Rugby |
| Swimming | Macumba | HIIT | Volleyball |
| Total Body | Martial Arts | Home Workout | Water Polo |
| Treadmill | Tennis | Weightlifting |  |
| Trekking |  |  |  |
| Walking |  |  |  |

This table provides a comprehensive listing of various sports activities categorized based on the type of physical and skill requirements. Each category represents a distinct aspect of physical fitness and expertise, ranging from endurance and strength to coordination and team collaboration. This classification was utilized to analyze and understand the sports preferences among different genders in the study.
